# 1429. Temperature and Humidity Variations in the Operating Room: A Risk Assessment

**DOI:** 10.1093/ofid/ofad500.1266

**Published:** 2023-11-27

**Authors:** Shauna Usiak, Judy Yan, Michell Reyes, Ann Martin, Tania N Bubb

**Affiliations:** Memorial Sloan Kettering Cancer Center, New York, New York; Memorial Sloan Kettering Cancer Center, New York, New York; Memorial Sloan Kettering Cancer Center, New York, New York; Memorial Sloan Kettering Cancer Center, New York, New York; MSKCC, New York, New York

## Abstract

**Background:**

The American Society of Heating, Refrigeration, and Air-Conditioning Engineers (ASHRAE) recommends that hospital operating rooms (OR) are controlled for temperature and humidity with established ranges but does allow for exceedances for surgeons and surgical procedures. There is currently no clear consensus or established thresholds for out-of-range temperature and humidity ranges in relation to risk for surgical site infections (SSI). A study was performed at Memorial Sloan Kettering Cancer Center (MSKCC) to further examine if temperature and humidity deviations pose a patient safety risk.

**Methods:**

The study included 5600 surgical procedures that were performed at MSKCC between September 2021 and January 2022 where temperature and humidity logs were available. Procedures were broken into groups based on in- (n=4419) and out-of-range (n=1187) temperatures and in- (n=5225) and out-of-range (n=381) humidity. SSIs following the procedure were identified using targeted positive microbiology cultures and within NHSN (National Healthcare Safety Network) surveillance periods. Chi-square and Fisher’s exact statistical tests were used to determine if there was a significant difference between groups where *p < 0.05*.

Breakdown of eligible procedures and follow up

**Figure:**
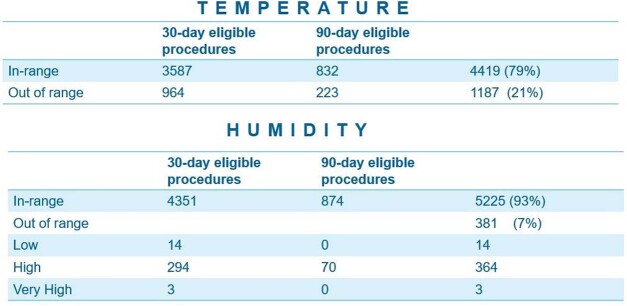

Table represents the total number of eligible procedures with environmental parameters and SSI follow up period.

**Results:**

There was no significant impact on SSI for out-of-range humidity (*p=0.15*). There was a significantly higher SSI rate observed for out-of-range temperatures (*p=0.04*) more specifically low out- of-range temperatures (*p=0.02*). A sub-analysis was performed for low temperatures based on procedures by duration. Significance of the finding was lost when surgery duration was 4 hours or greater.

**Conclusion:**

If there was a true correlation between SSI and low ambient temperature, we would have expected to see a strengthening of SSI risk with longer procedures. This study suggests that temperature and humidity deviations out of the recommended guidelines in the operating room do not pose additional risks for surgical site infections and limited exceedances can be permitted without risking patient safety.

**Disclosures:**

**All Authors**: No reported disclosures

